# Satisfactory clinical results and low failure rate of medial collagen meniscus implant (CMI) at a minimum 20 years of follow-up

**DOI:** 10.1007/s00167-021-06556-1

**Published:** 2021-04-09

**Authors:** Gian Andrea Lucidi, Alberto Grassi, Belal Bashar Hamdan Al-zu’bi, Luca Macchiarola, Piero Agostinone, Maurilio Marcacci, Stefano Zaffagnini

**Affiliations:** 1 IIa Clinica Ortopedica e Traumatologica, IRCCS Istituto Ortopedico Rizzoli, via Cesare Pupilli 1, 40136 Bologna, Italy; 2 Humanitas Clinical and Research Center-IRCCS, Via Manzoni 56, 20089 Rozzano, MI Italy

**Keywords:** Collagen meniscus implant, CMI, Meniscus scaffold, Post-meniscectomy, Long-term follow-up

## Abstract

**Purpose:**

The aim of the study was to evaluate the long-term clinical results, reoperations, surgical failure and complications at a minimum of 20 year of follow-up of the first 8 medial CMI scaffolds implanted by a single surgeon during a pilot European Prospective study.

**Methods:**

Seven (88%) out of 8 patients were contacted. The Cincinnati Score, VAS, and Lysholm score were collected. Moreover, magnetic resonance imaging (MRI) was performed on 4 patients at the last follow-up. Complications, reoperations and failures were also investigated.

**Results:**

The average follow-up was 21.5 ± 0.5 years. One patient underwent TKA after 13 years from CMI implantation; a second patient underwent valgus high tibial osteotomy 8 years after the index surgery and another patient underwent anterior cruciate ligament hardware removal at 21 years of follow-up. At the final follow-up, 3 patients were rated as “Excellent”, 1 as “Good” and 2 as “Fair” according to the Lysholm score. The Cincinnati score and the VAS were substantially stable over time. The MRI showed a mild osteoarthritis progression in 3 out of 4 patients according to the Yulish score, and the CMI signal was similar to the mid-term follow-up revealing 3 cases of myxoid degeneration and 1 case of normal signal with reduced scaffold size.

**Conclusion:**

The medial CMI is a safe procedure: satisfactory clinical results and a low failure rate could be expected even at a long-term follow-up. For this purpose, the correct indication as well as correcting axial malalignment and addressing knee instability at the time of the index surgery is mandatory. On the other hand, a mild osteoarthritis progression could be expected even after meniscus replacement.

**Level of evidence:**

IV.

**Supplementary Information:**

The online version contains supplementary material available at 10.1007/s00167-021-06556-1.

## Introduction

Over the last years, several research efforts have demonstrated the essential role of the meniscus for long-term knee function [31]. It is now fully appreciated that even partial meniscectomy increases the probability of developing osteoarthritis and accelerates the degeneration in joints with pre-existing chondropathy [18]. Persson et al. in a 10-year registry study, reported an absolute incidence of knee OA of 17% after partial meniscectomy compared with only 2.3% in the general population [19]. Even if large clinical trials reported the catastrophic long-term effect of meniscus resection, meniscectomy is still the most performed meniscus surgery [8, 10].

In fact, in cases of lesions in the white–white zone, poor tissue quality as well as complex or dislocated tears, meniscectomy could be the only possible treatment.

A subgroup of those patients will experience swelling, untreatable knee pain and tibial bone-marrow edema, a constellation of symptoms known as “post-meniscectomy syndrome”. In these circumstances, replacement of the meniscal tissue has been proposed as an effective treatment. While meniscus allograft transplantation (MAT) is indicated in cases of total meniscectomy, for partial resection, a meniscus scaffold could be implanted. Two artificial scaffolds are currently available for clinical use: the collagen meniscus implant (CMI), derived from bovine Achilles tendon, and the Actifit polyurethane scaffold. Since the first safety trials performed in animals [7, 25], meniscus scaffolds have gained attention because of the possibility to treat partial meniscus resection, a condition that does not represent an appropriate indication for MAT. Moreover, potential disease transmission and the reduced availability of allografts have contributed significantly to the development of these devices. The first clinical series of CMIs was published in 1997 [26], while the Actifit was later developed and the pilot trial refers to 2011 [29]. Since then, many clinical trials at a short- or mid-term follow-up have been published and most of them reported satisfactory outcomes after CMI surgery. However, only two of them reach the 10 years of follow-up [14, 35].

The purposes of this study were to present the long-term clinical results, surgical failure and complications at 21.5 years of follow-up of the first 7 medial CMI scaffolds implanted by a single surgeon during a pilot European Prospective study. The results of the present case series could be useful to set patients’ expectations in terms of clinical scores, reoperations, failures, and osteoarthritis progression at a long-term follow-up.

## Materials and methods

The study was conducted according to the principles of the Declaration of Helsinki. Approval of the study was obtained from the Institutional Review Board (IRB) of the “Casa di Cura Toniolo” (Prot. Gen. n.ro P360) and the “Istituto Ortopedico Rizzoli” (Prot. Gen. n.ro 0013050). Informed consent complied with European Union laws and was signed by the patient before enrollment.

### Patients cohort

The long-term clinical outcomes of the first 8 consecutive patients that underwent medial CMI implantation for medial meniscus defect between September 1997 and January 1999 at the Rizzoli Orthopaedic Institute were investigated. Although the procedure was indicated for both men and women, all the patients included in the study were male. The patients included in the present study represent a prospective cohort, where the short- and mid-term outcomes were reported in a previous publication [33]. According to the original experimental protocol, inclusion criteria for CMI implantation determined in the European Multicenter Prospective Study were: (1) irreparable medial meniscus tear at arthroscopy or a previous significant loss of meniscus after a partial meniscectomy; (2) traumatic or degenerative loss of meniscus cartilage; and (3) stable knee or surgically stabilized at the time of the implantation procedure. Exclusion criteria were: (1) axial deviations; (2) Outerbridge grade IV chondral lesions [17, 22]; (3) inflammatory or systemic diseases; (4) collagen allergies; (5) autoimmune diseases; and (6) pregnancy. All eight patients gave their written informed consent before the intervention. Although the implant procedure was indicated for both men and women, all eight patients in our study group were men.

Patients were reviewed in May 2019 with a minimum of 20-year follow-up. One patient (12%) was not available for the long-term evaluation; therefore, 7 male patients were included.

### Surgical technique and postoperative protocol

The complete surgical procedure has been described in the original study [33]. Briefly, after arthroscopic confirmation of CMI indication, the meniscus was debrided according to the presence of acute tear or chronic defect. The anterior and posterior meniscal attachment points were trimmed square to accept the scaffold, and the blood supply was enhanced by making puncture holes in the peripheral rim with a Steadman awl. After determining the defect size and trimming of the scaffold, the latter was positioned inside the joint and sutured to the host meniscus remnant with standard inside-out 2–0 sutures.

Physical therapy was started on the first post-operative day, and continuous passive motion was immediately allowed from 0° to 60°. The passive ROM was increased to 90° after 4 weeks, and full ROM was allowed after two further weeks. Weight-bearing was not allowed during the first 6 weeks. All patients followed a rehabilitation protocol for 6 months until they returned to full unrestricted physical activity.

### Patient’s evaluation

According to the original study protocol, all patients were evaluated regularly in the immediate post-operative period, and clinical assessment was performed at 3, 6, 12, and 24 months and from 6 up to 8 years. The clinical examination was performed utilizing the subjective Cincinnati Knee Rating System (score range 120–420) [15, 16] and pain self-evaluation was measured on a visual analogical scale (VAS) graded from 0 to 10. The patients were contacted at a minimum follow-up of 20 years, and the same subjective scores plus the Lysholm score (range 0–100) were collected. Patients were also inquired regarding complications and reoperations on the same knee during the considered follow-up. Patients with partial or total scaffold removal, Unicompartmental Knee Arthroplasty (UKA), and Total Knee Arthroplasty (TKA) were considered surgical Failures (SF). Moreover, patients with “Poor” Lysholm Score (< 65 points) at the final follow-up were considered Clinical Failures (CF). Magnetic resonance imaging (MRI) examinations were performed at the last follow-up and 6–8 years after surgery and were compared with the original pre-operative images. The MRIs were examined to check for implant signal alterations, and the cartilage status was evaluated according to the Yulish score [32]. Because of the lower quality of the MRI performed from 1997 to 1999, the data regarding the cartilage status were double-checked with the intraoperative arthroscopic evaluation according to the Outerbridge classification [17].

Due to the small number of patients included in the study, no statistical analyses were performed.

## Results

The age at surgery of the 7 included patients was 33.8 ± 8.9; considering the average follow-up of 21.5 ± 0.5, their final age was 55.2 ± 8.9 (Table 1).

**Table 1:** Demographic and surgical characteristics, mean ± SD [range]

Number of patients	7
Sex (M/F)	7/0
Age surgery (years)	33.8 ± 8.9 [24.5–51.4]
Age follow-up (years)	55.2 ± 8.9 [46.1–73.2]
Follow-up (years)	21.3 ± 0.5 [20.3–21.7]
Previous surgery	3 None; 2 MM; 2 MM + ACL-R
Acute/chronic	3/4
Defect size (mm)	41.4 ± 17.0 [25.0–70.0]
Defect %	66% ± 27% [35%–90%]
Scaffold size (mm)	32.9 ± 4.9 [25.0–40.0]
Concomitant surgery	4 None; 2 ACL-R; 1 Rev ACL-R + MFC mfx
Failures	1 (TKA after 13 years)
Surgery during follow-up	1 HTO; 1 hardware removal

*M* male, *F* female, *MM* medial meniscectomy, *ACL-R* anterior cruciate ligament reconstruction, *Rev* revision, *MFC* medial femoral condyle, *mfx* microfractures, *TKA* total knee arthroplasty, *HTO* high tibial osteotomy

**Fig. 1:**
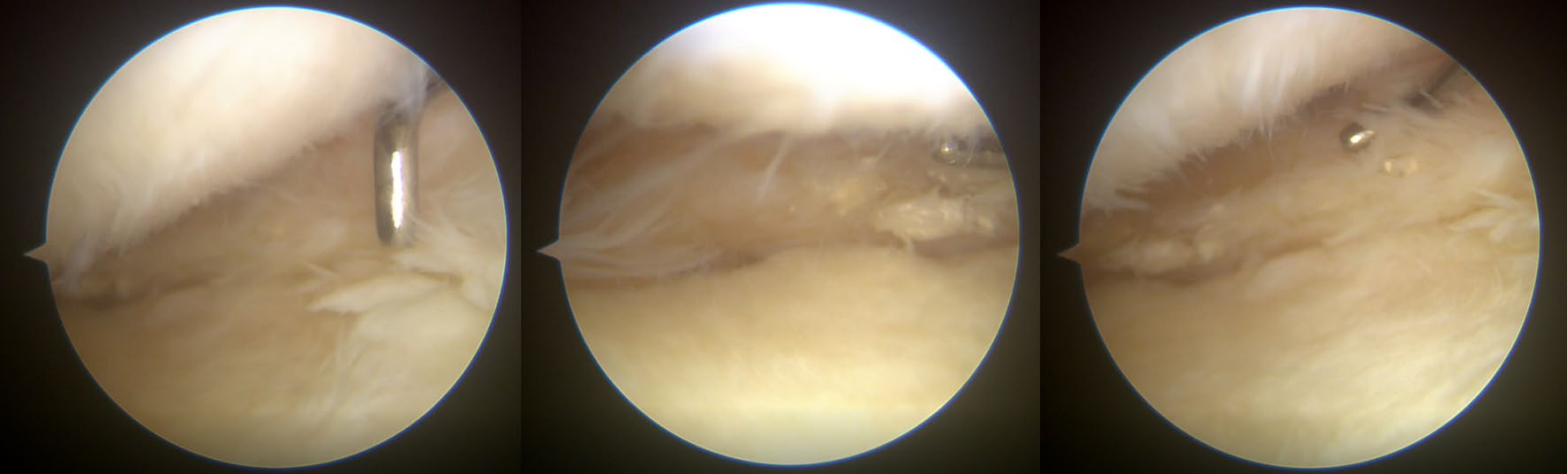
Arthroscopic images of a second look of the patient 7. This patient underwent partial medial meniscectomy 37 years ago at the age of 21. In 1999 the patient underwent ACL reconstruction combined with medial CMI implantation. During the surgery, a chondropathy grade II was already reported. There was evidence of mild cartilage degeneration at the last follow-up, while the CMI showed good integration with the host tissue and no tears were detected

During the 20 years of follow-up, only one patient underwent TKA after 13 years from CMI implantation at the age of 64. This patient's MRI revealed a reduction of scaffold size and fragmentation starting from 5 years after the index surgery. Another patient underwent valgus high tibial osteotomy (HTO) 8 years after CMI implantation due to knee swelling and progression of the varus deformity; this patient was not considered a failure, because CMI was not removed. Finally, one patient gave his consent to undergo a second-look arthroscopy and anterior cruciate ligament (ACL) hardware removal at 21 years of follow-up (Fig. 1).

**Fig. 2:**
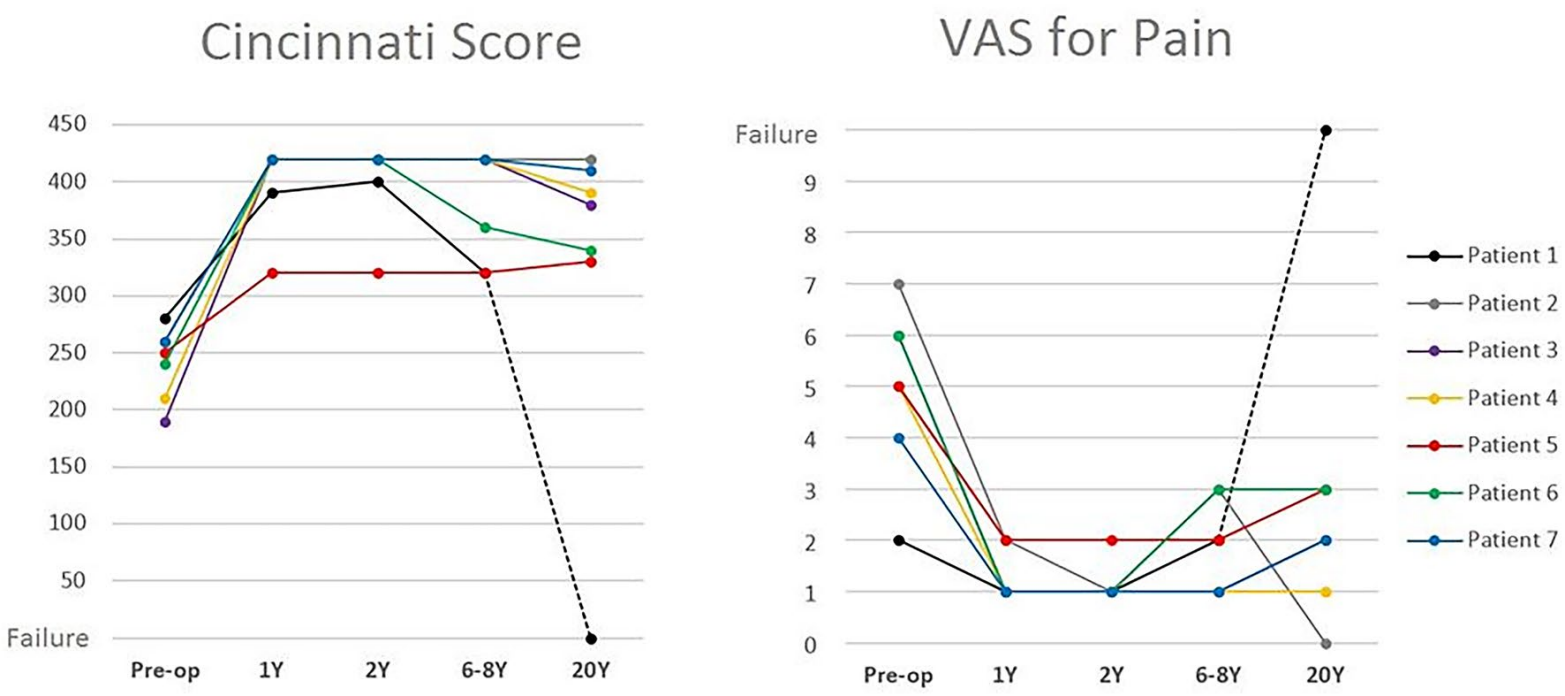
Cincinnati Score and the VAS for pain of the single patients are shown in the figure. Note that the Cincinnati score decreases slowly from the last follow-up and the VAS slightly increases

Of the 6 patients that were not considered failures, 3 were rated as “Excellent” according to the Lysholm score, 1 as “Good” and 2 as “Fair”. The Cincinnati score and the VAS for pain were substantially stable with respect to the previous follow-up (Fig. 2).

**Fig. 3:**
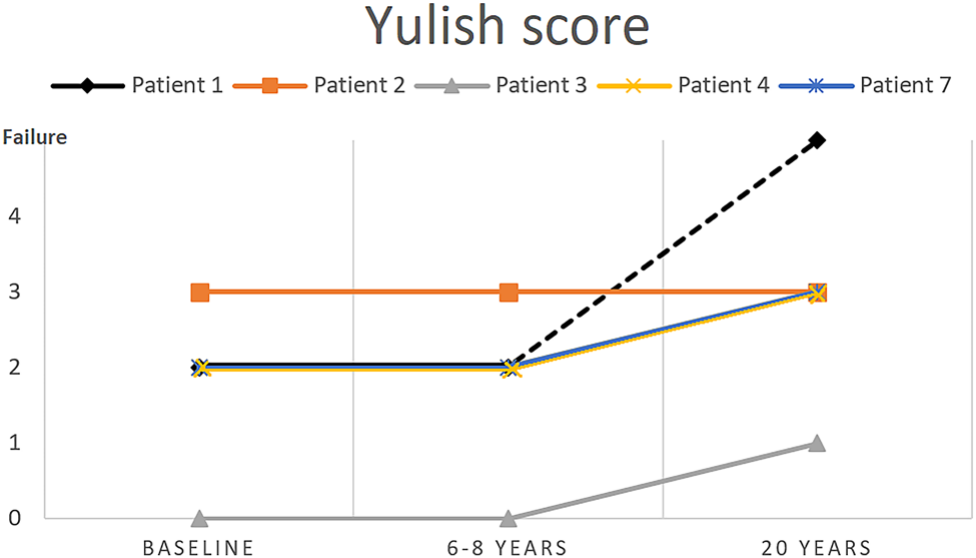
Yulish score of patients whose MRI was available at 20 years of follow-up. Note that the three with a better score had a slight worsening, while the patient with the worst score was stable over time

**Fig. 4:**
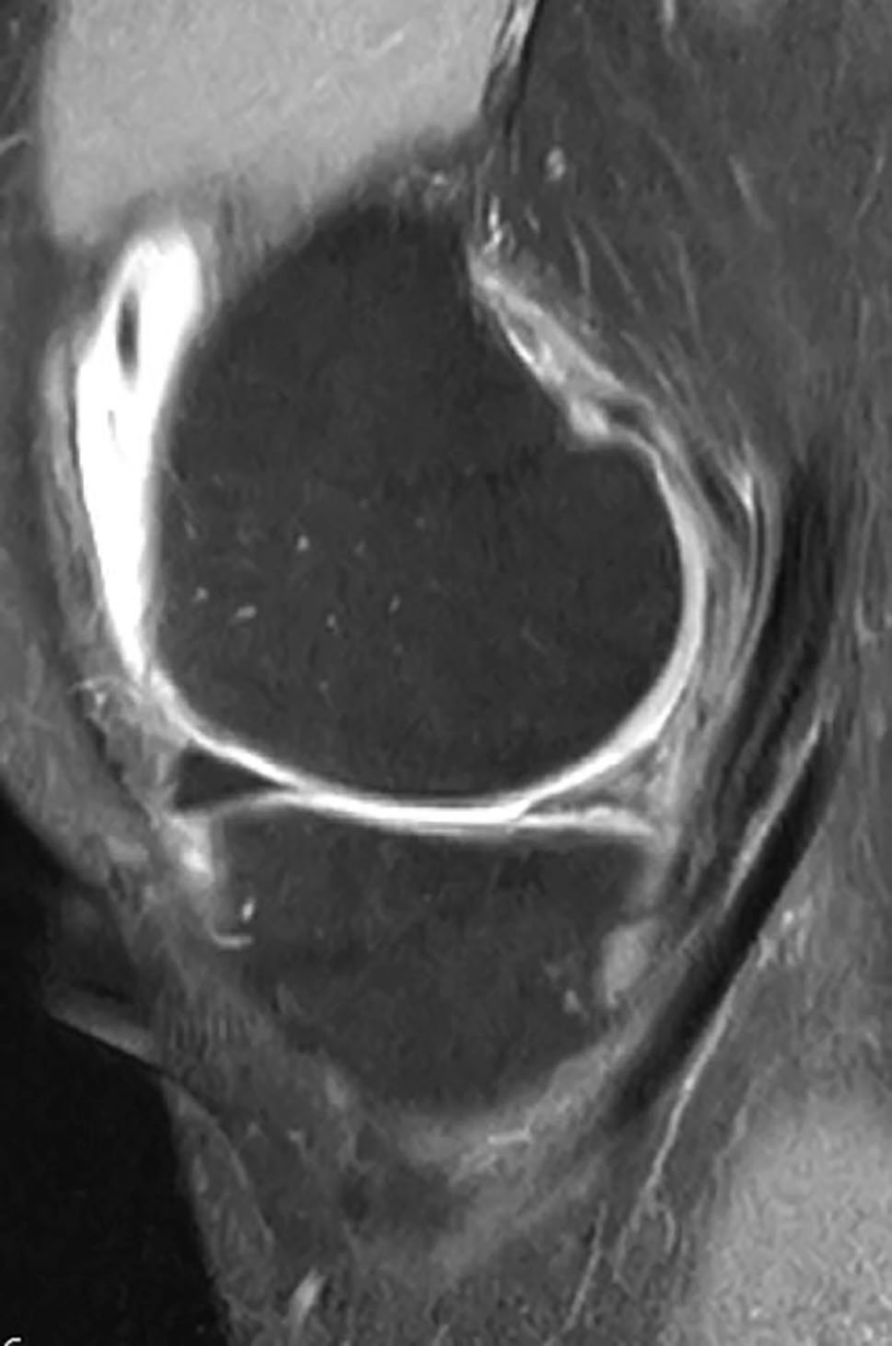
Sagittal MRI of patients 3 at 20 years of follow-up. Note that the implant is still recognizable and showing a good signal with reduced scaffold size

At the final follow-up, the osteoarthritis progression and scaffold signal of 5 patients were evaluated, including the patient that was considered a failure. The cartilage status showed no substantial difference in the first 6.7 years of follow-up in all the patients. Differently, from the mid term to the last follow-up, in 2 patients, a progression of the cartilage damage was noted, and one failure was recorded (Fig. 3). The CMI signal at the last follow-up was similar to the one recorded at the mid-term evaluation with 3 cases of myxoid degeneration and 1 case of normal signal with reduced size of the scaffold (Fig. 4).

## Discussion

The main findings of the study were that the CMI implant for partial meniscal resection could provide pain relief and good knee function at a minimum of 20-year follow-up. However, the decision not to address at the time of the index surgery all the underlying knee pathology could result in suboptimal clinical outcomes at a mid-term follow-up and additional surgeries or implant failure at the long term.

The indications for MAT or meniscal scaffold are nowadays comprehensively defined. While the MAT is the procedure of choice in the presence of total or subtotal meniscal resection, the CMI could be implanted if partial meniscectomy was previously performed [5]. The analysis of the only failure of our series further stresses these indications. The patient was the older of our series (51 years) with a chronic medial meniscal deficiency after a medial meniscectomy performed 10 years before. At the time of surgery, a grade II chondropathy was noted, and a defect size of 70 mm was measured in the medial compartment. Unfortunately, it was only possible to fill the defect with an undersized 40 mm scaffold. Interestingly, the clinical scores improved significantly and remained excellent in the first 2 years after surgery, while a progressive decrease of the PROMs was recorded at 6.8 years of follow-up. A subsequent rapid worsening of the symptoms was noted at a longer follow-up and a TKA was implanted 13 years after the index surgery.

In the present study, five patients underwent serial MRI evaluation to assess the cartilage degeneration during the follow-up period. Excluding the only failure that was previously discussed, 3 out of 4 patients experienced a mild progression of cartilage degeneration at the long-term follow-up [34].

Verdonk et al. [28] evaluated a series of 41 patients that underwent MAT at a mean of 12.1 years of follow-up. Excluding the patients that underwent TKA, the MRI analyses showed the progression of cartilage degeneration in 11/17 knees (65%) according to the Yulish score. Thus, suggesting that some degree of cartilage degeneration must be expected in the presence of symptomatic meniscus deficient compartment. Similarly, Toanen et al. reported a progression of cartilage degeneration in 38.9% of the patients after Actifit scaffold implantation at 5-year follow-up. [27]

While dealing with medial meniscus replacement surgeries, combined procedures are recommended if varus deformity, ligament instability, or focal chondral defect are present [5, 6]. If not corrected, the axial malalignment entails abnormal pressure on the MAT, thus causing reduced vascularization, extrusion, loosening, and degeneration of the graft [11, 20]. These mechanical principles could be easily applied to the CMI, since it acts as a three-dimensional scaffold that should be colonized by host cells and vessels to promote the formation of functional tissue [22].

Histological studies showed that 1 year after implantation, 75–90% of the CMI is replaced by host meniscus-like tissue and that the scaffold is expected to be reabsorbed in 12–18 months. Therefore, the joint homeostasis in this first period seems crucial for the correct maturation of the scaffold [3, 21]. In one patient, a varus malalignment of 4° was tolerated and not corrected at the time of the CMI implantation. After 2 years, the patients' MRI showed a gross decrease in size without a clear implant-capsule junction, thus demonstrating a poor integration with the host tissue. During the follow-up period, the patient experienced a progression of the varus deformity and worsening of the symptoms and underwent an osteotomy 8 years later. At the last follow-up, the fair clinical scores suggest that, in retrospect, a more aggressive surgical approach could have been the right decision in this case.

The menisci and the ACL are interdependent for the antero-posterior knee stability: the medial meniscus acts as a secondary stabilizer and its role becomes even more crucial in the ACL-deficient condition [30]. Therefore, it is highly recommended to associate an ACL reconstruction to a MAT or a meniscus scaffold if some degree of instability is reported. In our series, 3 patients underwent an ACL-reconstruction or revision at the time of the CMI implantation and another patient had a successful ACL reconstruction 10 years before. Of these 4 patients, none underwent subsequent surgeries or experienced CMI failures and all except one present good or excellent Lysholm at 21.5 years of follow-up. Similarly, Toanen et al. [27] reported superior clinical outcomes in patients that underwent combined ACL reconstruction compared with those who underwent a single scaffold implantation [27].

It is difficult to compare the results of our series with the literature, since only two series of CMI have been published with long-term results and both are limited to a 10 years of follow-up. Monnlau et al. [14] reported the outcomes of 25 patients treated with CMI implantation: among these patients, 5 presented chronic meniscal defects and 20 were treated in acute because of large and irreparable meniscal tears. The mean Lysholm scores of these patients improved from 59.9 preoperatively to 89.6 at 1 year. They remained at similar values of 87.5 at a minimum of 10-year follow-up, demonstrating a constant trend over time. In our series, a mild reduction of the Cincinnati Score is present in 5 out of 7 patients when comparing the different follow-up of 6.8 and 21.5 years. Similar to our series, Monnlau reported a failure rate of 8% (2 of 25), with two patients requiring MAT [14].

Therefore, the procedure proved to be safe with a low rate of implant failure even at a long-term follow-up. In a large multicentre prospective clinical trial of 311 patients, the medial meniscectomy was compared to the medial CMI [21]. Interestingly the reoperation rate at 5 years was 2.7 times greater in the meniscectomy group. Hirschmann et al. [9] investigated the complication and reoperation rate of a series of 67 patients: 1 implant failure, 1 chronic synovitis and 1 infection were reported at 1-year follow-up. In the present study, no device-related complication such as chronic synovitis, late infections or immunological reactions were reported at 20-year follow-up, suggesting that the complication may be more frequent during the first years after surgery.

The present study has several limitations. First, the number of patients included in this study was very small. In fact, when the surgeries of this study were performed, only reports on animals and one clinical feasibility trial on humans were published [26]. Moreover, this pilot study included an heterogenous group of patients in terms of age at surgery, axial alignment, previous and associated procedures and chondropathy at the time of index surgery. Therefore, it is clear that the results of this paper could not be generalized and that studies with a larger sample size are needed to confirm the results pointed out by our research. On the other hand, this prospective paper presents the outcomes of 7 out of 8 patients that underwent several follow-ups and it is the first that reports the CMI results at more than 20 years after surgery.

A second limitation is that the osteoarthritis progression during the 21 years of follow-up was evaluated using different MRI scanner devices. However, to reduce the bias caused by lower quality images of the MRI performed in the preoperative period, those data were double-checked with the intraoperative cartilage findings according to the Outerbridge classification [17] which is very similar to the Yulish score used for the MRI evaluation.

Another limitation of the study is the lack of a control group of patients that underwent isolated meniscectomy to compare the clinical results of these two procedures and to analyze if the CMI implants really provide some advantages at a long-term follow-up in terms of chondroprotection, failures and reoperations.

Given all these limitations, the information in the present study could be useful in the clinical practice to set patients’ expectations in terms of clinical scores, reoperations, failures, and osteoarthritis progression during a long follow-up period after CMI implantation.

## Conclusion

The arthroscopic medial collagen meniscus implant (CMI) is a safe procedure: satisfactory clinical results and a low failure rate could be expected even at a long-term follow-up. For this purpose, it is crucial to perform a careful clinical and radiological evaluation and correct axial malalignment and knee instability at the time of the index surgery. On the other hand, a mild osteoarthritis progression could be expected even after meniscus replacement.

## Supplementary Information

Below is the link to the electronic supplementary material.

Supplementary file1. Arthroscopic second look 21 years after ACL reconstruction and medial CMI implant. Please note a partial reabsorption of the scaffold that was replaced with meniscus-like tissue (MP4 4995 KB)

## Declarations

### Availability of data and material

The authors declare that the data supporting the findings of this study are available within the article, additional data are available from the corresponding author upon request.
